# Advances in Receptor-like Protein Kinases in Balancing Plant Growth and Stress Responses

**DOI:** 10.3390/plants12030427

**Published:** 2023-01-17

**Authors:** Qingfeng Zhu, Yanzhao Feng, Jiao Xue, Pei Chen, Aixia Zhang, Yang Yu

**Affiliations:** Guangdong Key Laboratory of Crop Germplasm Resources Preservation and Utilization, Key Laboratory of South China Modern Biological Seed Industry, Ministry of Agriculture and Rural Affairs, Agro-Biological Gene Research Center, Guangdong Academy of Agricultural Sciences, Guangzhou 510640, China

**Keywords:** receptor-like protein kinases, growth, stress responses, crosstalk

## Abstract

Accompanying the process of growth and development, plants are exposed to ever-changing environments, which consequently trigger abiotic or biotic stress responses. The large protein family known as receptor-like protein kinases (RLKs) is involved in the regulation of plant growth and development, as well as in the response to various stresses. Understanding the biological function and molecular mechanism of RLKs is helpful for crop breeding. Research on the role and mechanism of RLKs has recently received considerable attention regarding the balance between plant growth and environmental adaptability. In this paper, we systematically review the classification of RLKs, the regulatory roles of RLKs in plant development (meristem activity, leaf morphology and reproduction) and in stress responses (disease resistance and environmental adaptation). This review focuses on recent findings revealing that RLKs simultaneously regulate plant growth and stress adaptation, which may pave the way for the better understanding of their function in crop improvement. Although the exact crosstalk between growth constraint and plant adaptation remains elusive, a profound study on the adaptive mechanisms for decoupling the developmental processes would be a promising direction for the future research.

## 1. Introduction

Life represents a contradictory unity of growth and stress response. During the growth, development, and reproduction of plants they may suffer from a variety of abiotic and biotic stresses, such as drought, salinity, cold, heat, toxic metals, and diseases [1,2]. To adapt to changes in the natural environment, plants carry out the simultaneous regulation of growth and stress through a series of signal transduction. In these regulatory pathways, receptor-like protein kinases (RLKs) have attracted widespread attention. Similar to the receptor protein kinases encoded by animal genomes, plants also contain many proteins homologous to animal receptor protein kinases, which are named plant receptor-like protein kinases.

The first plant RLK was found in corn [3], while RLK proteins have been reported in almost all plants to date. RLKs have a large family with at least 610 members in Arabidopsis and at least 1131 members in rice [4]. A typical RLK consists of a single transmembrane domain, an intracellular kinase domain, and a singular extracellular region that is considered to sense the external signal. Plant RLKs are originally sensors for a variety of signals like phytohormones and small peptides. They facilitate intercellular communication during plant growth, development, and stress response. For instance, BRASSINOSTEROID INSENSITIVE 1 (BRI1) binds BRI1-ASSOCIATED RECEPTOR KINASE 1 (BAK1) to perceive brassinosteroid signals and control various aspects of plant growth and development. One well-studied small peptide is CLAVATA1 (CLV1), which associates with CLAVATA3 INSENSITIVE RECEPTOR KINASES (CIKs) to sense the CLV3 signal and regulate meristem homeostasis. The RLK-mediated signaling circuit is one of the abiotic/biotic stress responses that plants use to adapt to the constantly changing environmental conditions. As an example, FERONIA (FER), a member of the CrRLK1L family, is essential for stress reactions. RLK-mediated signaling increases the transcriptional activation of several defense and pathogenesis-related genes to thwart the pathogens’ attack and neutralize the damage they cause. The flagellin sensitive 2 (FLS2) preferentially recognizes the bacterial flagellin epitope (flg22) to initiate the recruitment of coreceptors and following phosphorylation. FLS2 typically heterodimers with BAK1 and undergoes transphosphorylation. Overall, RLKs play critical roles in regulating both plant development and stress responses [5,6,7,8,9,10].

**Figure 1:**
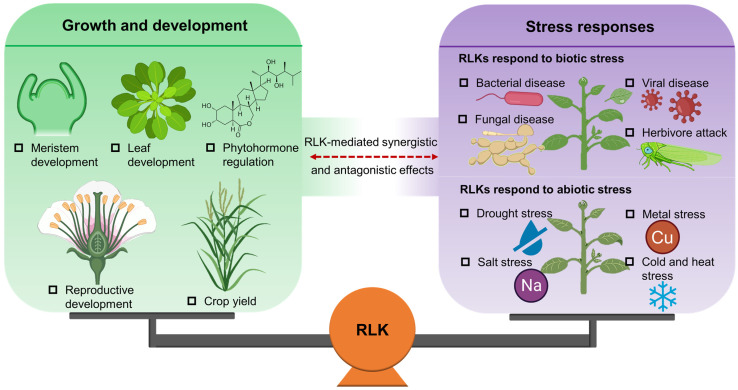
Schematic diagram of receptor-like protein kinase (RLKs) in balancing plant growth and stress responses.

The functional characterization of RLKs has emerged as one of the most exciting areas of plant biology. In recent years, research on the function and mechanism of RLKs has become a hot topic in the balance of plant growth and environmental adaptation (Figure 1). In this paper we systematically review the recent research progress on RLKs from four aspects: the classification of RLKs; the regulatory roles of RLKs in plant growth and development; the biological function of RLKs in plant stress response; and the molecular mechanism of RLKs that coordinated regulate plant growth and stress response, with a view to provide a reference for plant molecular biology and crop genetics and breeding.

## 2. Classification of RLKs

Most plant RLKs have three important protein domains: the extracellular domain (ECLB); the transmembrane domain (TM); and the cytoplasmic kinase domain (CKD). The ECLB is located at the N-terminus and is connected with the signal peptide (SP). It can sense an external signal, polymerize with homologous or heterologous partners, and then start the signal transmission process. The TM is located on the cytoplasmic membrane and contains 22–28 amino acids. It can link internal and external structures together and is responsible for fixing specific proteins on the membrane and internal and external signal transmission. The C-terminal CKD domain consists of a protein kinase catalytic domain and a juxtamembrane region. The protein kinase catalytic domain is highly conserved, with serine/threonine phosphorylation sites, and transmits signals downstream through phosphorylation. The juxtamembrane region can separate the transmembrane structure from the kinase region [11].

**Figure 2:**
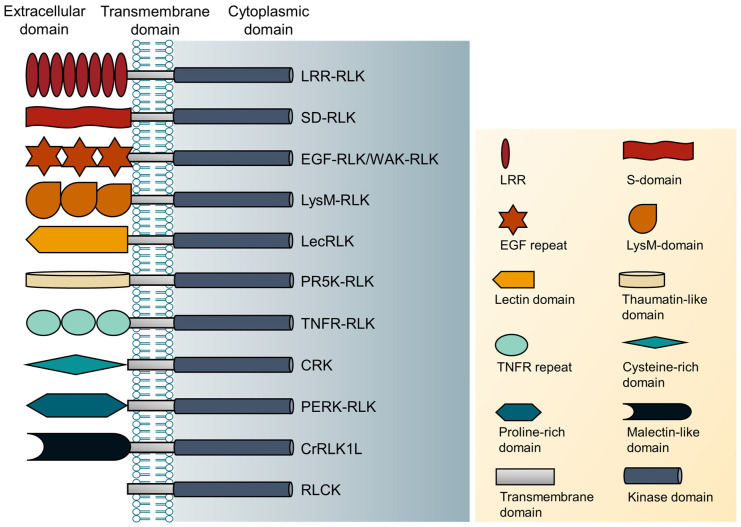
Protein domain architectures of 11 selected RLKs.

There are many types of extracellular domains of plant receptor-like protein kinases. According to their characteristics, RLKs can be divided into at least eleven subfamilies [9] [9] (Figure 2, Table 1). In addition to the typical RLKs, there are also a large number of atypical receptor-like protein kinases in plants. Receptor-like cytoplasmic kinases (RLCKs) are RLKs that are fixed on the plasma membrane but lack extracellular binding domains. RLCKs play an important role in plant immunity, stress response, and growth and development [12,13].

## 3. Regulation of RLKs on Plant Growth and Development

The complicated integration of diverse environmental cues and growth information that results in plant development is a highly regulated process that requires the coordinated actions of numerous pathways [14,15,16,17,18]. RLKs are constantly added to or removed in the entire life cycle, which forces plants to control their development in order to adapt to various environments. However, because plants vary in spatial and temporal patterns, the impacts of RLKs on plant growth and development are more complicated.

### 3.1. Meristem Development

Plant meristems, which function as repositories of pluripotent stem cells, are essential for the continuous initiation and development of new organs. Several RLK-mediated signaling pathways that are involved in preserving meristem homeostasis have been identified in recent years. For example, CLV1 was found to be a member of the LRR-RLK subfamily in *Arabidopsis thaliana*. Studies have shown that CLV1 is related to meristem activity [19,20]. CLV1 can sense the small peptide CLV3, and then inhibit the expression of WUSCHEL (WUS). WUS is an important homeodomain transcription factor, which can diffuse to the central region of shoot apical meristem (SAM) and promote the expression of CLV3. Research shows that CLV1 recruits CIKs to form a receptor/co-receptor complex, and subsequently mediates CLV3-WUS feedback loop signals and controls the size of flower meristem and the number of flower organs (Table 2) [21]. However, it is still unclear why CLV3 expression is specifically restricted to the stem cell by the WUS-STM module [22]. CIKs are a group of novel co-receptor protein kinases that control stem cell homeostasis. They can act as co-receptors for CLV1, RECEPTOR-LIKE PROTEIN KINASE2 (RPK2), and BARELY ANY MERISTEM1/2 (BAM1/2) and play an important role in regulating CLV3-mediated stem cell homeostasis through phosphorylation-mediated CLV3 signals [23,24]. Given that these RLKs are conserved in plants, the above observations reaffirm that RLK-mediated signaling operates meristem development and may be prevalent and diverse in plants.

### 3.2. Leaf Development

The RLK family contains several proteins that have been identified as leaf development regulators. The ERECTA family (ERf) belongs to the LRR-RLK subfamily, including ERECTA (ER), ERECTA-LIKE1 (ERL1), and ERL2. They play important roles in regulating leaf morphology and stomatal development and responding to biotic and abiotic stresses in Arabidopsis. EPIDERMAL PATTERNING FACTOR-LIKE2 (EPFL2) and EPFL9 belong to the cysteine-rich secretory peptides in the EPIDERMAL PATTERNING Factor-Like (EPFL) family. EPFL2 interacts with ERf-RLK to promote cotyledon growth [25]. EPFL2/9 cooperates with ERf-RLK to help coordinate the ovule pattern, so as to coordinate the number of seeds and the growth of pistils and fruits [26]. OsERL is a homologous isomer of rice ERf and plays a role in the establishment of anther lobes [35]. These findings show that the manipulation of RLK expression is crucial for controlling both leaf morphology and senescence.

### 3.3. Reproductive Development

Emerging evidence indicates that RLKs play essential roles in plant reproductive development. BAM1/2 and RPK2 belong to the LRR-RLK subfamily and are required for early anther cell development. Research shows that, as in the case of CLV1, BAM1/2 and RPK2 form receptor and co-receptor complexes by recruiting CIKs, mediate CLV3-WUS feedback loop signals, regulate the division of sporogenous cells, and determine the size of anther wall cells [21]. AtVRLK1 (Vascular-Related Receptor-Like Kinase 1) belongs to the LRR-RLK subfamily, which is expressed specifically in cells underlying secondary cell wall thickening and the upregulation of this gene affects anther dehiscence [27]. AtPERK5 (Proline-rich extensin-like receptor kinases 5) and AtPERK12 participate in the growth of pollen tubes. The study found that the pollen tube growth in single and double knockout mutants of *perk5-1* and *perk12-1* was damaged with defective male gametophytes, and excessive pectin and cellulose accumulation was shown on the cell wall of the pollen tube [28]. Thermo-Sensitive Genic Male Sterile 10 (TMS10) and TMS10-LIKE (TMS10L), members of the rice LRR-RLK subfamily, are related to tapetal degeneration and pollen vitality [36]. OsLecRK5 can phosphorylate the callose synthase UDP glucose pyrophosphorylase 1 (UGP1) during the meiosis of rice anther microspore mother cells and promote its activity in callose biosynthesis [37]. These results demonstrate a molecular relationship between RLKs and plant reproductive development. Nevertheless, present research has little known about the molecular mechanisms of how RLKs act to modulate plant reproduction. Identification substrates of RLKs in reproductive organs will be a helpful strategy to solve this problem.

### 3.4. Crop Yield

The discovery of the genetic basis of the desired quantitative trait is necessary for effective breeding utilizing genetic engineering approaches. Only a small number of RLKs influencing these traits have been cloned and described due to the genetic complexity of crop yield traits. The plants overexpressing OsLSK1 (Large spike S-domain receptor-like Kinase 1) extracellular domain and transmembrane domain showed higher culm, larger spikelet, and higher yield [38]. OsER1 encodes ERECTA1, which negatively regulates the number of grains per spike and acts on the upstream of OsMKK10-OsMKK4-OsMPK6 signal pathway, and it controls the number of spikelets by regulating cytokinin metabolism in rice [39]. The overexpression of wheat *TaBRI1* gene in Arabidopsis resulted in earlier flowering time and higher seed yield [43,44,45]. Even though these RLKs are crucial in controlling agricultural yield components, only a few of their functions in crop yield traits have been reported to date.

### 3.5. Phytohormone Regulation

**Figure 3:**
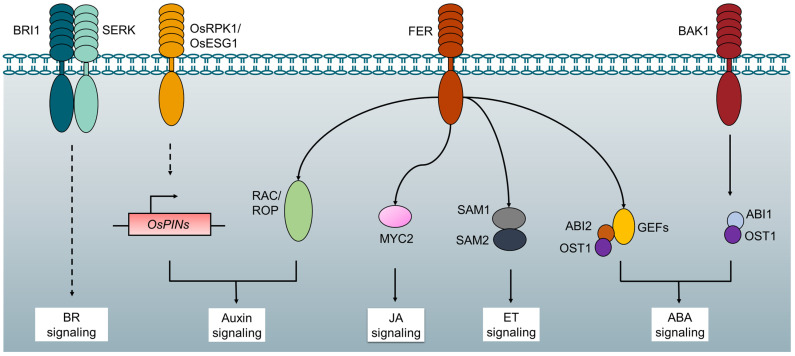
RLKs regulate phytohormone signaling. BRI1 recruits its co-receptor SERK3/BAK1 in the process of sensing BR signals. OsRPK1 and OsESG1 affect transport and distribution of auxin. FER interact with ROP/RAC, MYC2, SAM1/SAM2, GEFs/ABI2/OST1 to regulate auxin signaling, JA signaling, ET signaling and ABA signaling, respectively. ABI1 interacts with BAK1 to cause ABA-induced stomatal closure.

RLKs are excellent candidates of receptors for phytohormone-inducing signals because they function by detecting signals at the cell surface and transmitting those signals across the plasma membrane to trigger signal transduction inside the cell (Figure 3). BRI1 is a typical LRR-RLK, which is the main receptor of BR and regulates normal plant growth and cell elongation by mediating BR signaling [29,30,31,32,33]. BRI1 usually recruits its co-receptor BAK1 in the process of sensing BR signals. BAK1 is also named SOMATIC EMBRYOGENESIS RECEPTOR KINASE 3 (SERK3) [46]. BAK1 and SERKs act as co-receptors of various RLK receptors to regulate various growth and development processes of plants [47]. The BR signaling pathway mediated by the BRI1-SERK complex activates the BRI1-EMSSUPPRESSOR1 (BES1) family transcription factors by recruiting RLCKs, protein phosphatases, and Glycogen synthase kinase 3 (GSK3)/Shaggy-like kinase, directly regulating the expression of BR response genes, thus regulating multiple aspects of plant growth and development [33,48]. The BRI1-SERK and Excess Microsporocytes 1 (EMS1)-SERK signaling pathways recruit the same BES1 family transcription factors to control cell elongation and anther development, respectively [34,49]. SERK2 is a component of rice BR signal and has been reported to regulate rice BR signal and salt tolerance, overexpression of SERK2 significantly enhances grain size and salt stress resistance [40].

In addition to regulating BR pathways, a number of RLK proteins have been characterized for their roles in the signaling of other plant hormones [50,51,52,53,54,55,56,57,58]. Auxin-regulated root hair growth is mediated by FER, which works in the RAC/ROP signaling pathway [59]. The loss-of-function of *OsRPK1* promote the growth, plant height, and tiller number rice. On the contrary, the overexpression of *OsRPK1* can cause adventitious roots, underdeveloped lateral roots, and reduced root apical meristem. Research shows that OsRPK1 affects root structure by negatively regulating auxin transport in rice [41]. OsESG1 can regulate the initiation and development of crown and root by controlling the response and distribution of auxin, and it can participate in drought stress response by regulating antioxidant activity and stress response gene expression [42]. ABI1 interacts with BAK1 and prevents BAK1 and OST1 from interacting to cause ABA-induced stomatal closure in guard cells [60]. FER has multiple roles in phytohormone signal transduction. Through the activation of ABI2, which FER negatively regulates, GEFs cooperate with FER and ROP11/ARAC10 to convey the FER signal in a manner that is responsive to ABA [61]. Through interactions with SAM1 and SAM2, FER inhibits the synthesis of SAM, which in turn negatively affects the formation of ethylene [62]. In jasmanic acid (JA) signaling, FER phosphorylates and destabilizes the transcription factor MYC2 [63]. Since FER plays an important role in phytohormone response, studying the transcriptional and translational regulators of FER will promote our knowledge of how RLKs orchestrate different hormone signals. 

## 4. Biological Functions of RLKs in Plant Stress Response

### 4.1. RLKs Respond to Biotic Stress

When plants are attacked by pathogens, RLKs conduct defense response to invading pathogens or external signal molecules on the plasma membrane [64,65,66,67]. This paper briefly describes RLKs and their functions involved in biological stress response, as reported in recent years (Table 3).

#### 4.1.1. Bacterial Disease

Recent studies have shown that the negative regulation of CONSTITUTIVE DIFFERENTIAL GROWTH1 (CDG1) in PTI is independent of the BR signal. Arabidopsis CDG1 can interact with FLS2 and CHIN elicitor receptor kinase 1 (CERK1), and negatively regulate the immunity induced by flg22 and chitin by promoting the degradation of FLS2 and CERK1. In addition, the effector of *Pseudomonas syringae* AvrRpm1 can induce interaction between CDG1 and its host target RPM1 interaction PROTEIN4 (RIN4), phosphorylating RIN4 at multiple sites. Therefore, knocking out of *CDG1* gene can reduce the plant allergic reaction induced by AvrRpm1 and increase the growth of AvrRpm1-secreting bacteria in plants [68]. Arabidopsis *SERK1* and *SERK2* are important genes for resistance to bacterial leaf blight and fungal infection [69]. *CRK28* and *CRK29* act on pathogen perception and enhance plant immune response [70]. By screening rice RLK mutants infected by *Xanthomonas oryzae* pv, a gene *rrsRLK* (required for ROS scarifying receptor-like kinase) encoding RLCK was identified. The *rrsRLK* mutant showed resistance to a variety of *Xoo* strains, and showed abnormal peroxisome biosynthesis, hydrogen peroxide (H_2_O_2_) accumulation, and reduced reactive oxygen species (ROS) scavenging ability [71,74]. Nevertheless, we know nothing about the reasons why the activity of ROS scavenging enzymes is changed.

#### 4.1.2. Fungal Diseases

A new rice blast resistance gene *Pi65* was cloned from resistant variety GY129, which encodes LRR-RLK. Overexpression of *Pi65* enhances rice blast resistance [75]. The loss-of -function mutant *SDS2* (SPL11 cell death suppressor 2) shows attenuated immune response and increased susceptibility to rice blast. The overexpression of *SDS2* induces programmed cell death, accompanied by an enhanced immune response and enhanced resistance to rice blast fungus. In addition, SDS2 interacts with OsRLCK118 and OsRLCK176, which stimulate the production of activated oxygen by phosphorylating OsRbohB, thus positively regulating the immune response [76]. It is reported that WAKs play different roles in blast resistance in rice. OsWAK14, OsWAK91 and OsWAK92 confer resistance to rice blast, but OsWAK112d increases the sensitivity to blast fungus [82]. However, the molecular signals downstream these WAKs is yet to be identified. These observations suggest that opposite roles of RLKs in the same family. FER, which belongs to Catharanthus roseus family, leads to increased susceptibility to powdery mildew [83]. AtBAK1 is reported to be involved in the process of plant basic immune response [71]. *TaXa21*, highly homologous to *Xa21*, has been cloned from wheat, which interacts with TaWRKY76 and TaWRKY6 and confers the resistance to stripe rust in wheat seedlings under high-temperature conditions [80]. *TaCRK10* is a CRK gene identified from wheat, which also confers resistance to stripe rust under high temperature similar to *TaXa21* [81]. Although the gene function of TaXa21 resembles TaCRK10, their molecular targets are different, indicating that plants have evolved different pathways to cope with the same pathogen, which may be an advantage when one of the pathways is suppressed by the fungus.

#### 4.1.3. Viral Disease

It is interesting to note that, in recent years, numerous studies have shown that various RLKs affect plants’ sensitivity to viruses and, in some circumstances, interact with plant or viral proteins. OsSOBIR1 acts as a receptor kinase and combines with OsRLP1 to form a signal receptor complex, triggering PTI-related reactions and activating rice immunity against rice black-streaked dwarf virus (RBSDV) infection [77]. Surprisingly, SOBIR1 homolog in tomato does not function in antiviral infection [84]. The ability of plant viruses to disseminate their genomes from an initially infected cell to nearby cells through a variety of pathways has allowed for both local and systemic virus dissemination in plants. A recent study found a direct contact between BAM1 and the *Tobacco Mosaic Virus* movement protein, indicating that BAM1 may play a role in the early phases of virus dissemination and the transportation of viral protein from cell to cell [72]. Crosstalk among RLK-mediated signal transduction pathways can occur when many pathogens are infected at the same time in the same host. It is reported that the inverse modulation of antiviral and antibacterial immunity is mediated by the receptor-like kinase NIK1, which may allow bacteria and viruses to stimulate host immunological responses against one another [73]. Therefore, it is of interest to further investigate the crosstalk between antibacterial and antiviral pathways.

#### 4.1.4. Herbivore Attack

It takes a very sophisticated mechanism for plants to defend themselves from herbivores [85,86,87]. Protein kinases play a key role in every stage of the host defensive response, from sensing to distal downstream signaling [88,89]. The rice OsLRR-RLK1 was upregulated when the plant was attacked by a striped stem borer (SSB) and treated with oral secretions of *Spodoptera frugiperda*. Further studies showed that OsLRR-RLK1 acts upstream of the mitogen-activated protein kinase (MPK) cascade and positively regulates defense-related MPKs and WRKY transcription factors. In addition, the levels of herbivore-induced jasmonic acid and ethylene can be positively regulated by OsLRR-RLK1, which in turn affects the activity of defense-related trypsin protease inhibitors and SSB resistance [78]. Oppositely, the same group reported a second leucine-rich repeat receptor-like kinase in rice, OsLRR-RLK2, which exhibited a negative role in the resistance of rice to brown planthopper [79]. These data offer a means of enhancing plant productivity and resistance to herbivores by identifying and modifying RLKs. However, the ligands of both OsLRR-RLK1 and OsLRR-RLK2 are uncharacterized, so further studies may help us know more about the herbivore–plant interplay.

### 4.2. RLKs Respond to Abiotic Stress

The growth and development of plants in nature are affected by various environmental changes such as drought, high salt, cold, and metal. Plants have evolved a variety of mechanisms to cope with environmental changes, among which RLKs play an important role in coping with environmental stimuli (Table 4).

#### 4.2.1. Drought Stress

Drought and salt, as the main abiotic stresses in plants, are related to the plant hormone ABA pathway. ABA is a key regulator of gene activation related to osmotic stress response under drought and salt conditions [102,103,104]. *Leaf Panicle 2* (*LP2*) encodes LRR-RLK, which can interact with aquaporins OsPIP11, OsPIP13, and OsPIP21 in drought response, and plays a role as a negative regulator in drought response by regulating active oxygen metabolism, stomatal density, and stomatal closure [93]. LRR-RLK protein HAESA-LIKE3 (HSL3) plays a role in regulating stomatal closure and drought tolerance. The excessive accumulation of H_2_O_2_ in the hsl3 loss-of-function mutant changed the transmembrane anion outflow in the protective cells, leading to stomatal closure and enhanced tolerance to drought stress [94]. RLK7, a plasma membrane-localized LRR-RLK, can recognize Arabidopsis secreted peptide PIP1. PIP1-RLK7 signal pathway induces stomatal closure by activating S-type anion channel SLAC1 (Slow Anion Channel1) [90,91]. RPK1 regulates ABA/stress signal by controlling homeostasis in ROS. In addition, RPK1 and BAK1 form complexes with Open Stomata 1 (OST1), a member of the cytoplasmic sucrose non-fermenting 1-related sub-family 2 (SnRK2) protein kinases/SnRK2.6 sub-families, to regulate ABA-induced stomatal closure [92,105,106]. Interestingly, recent study shows that OST1 may involve in fruit ripening, a process that requires moderate drought [107]. Future research may reveal the balance between drought and plant growth modulated by OST1. Therefore, OST1 has the potential to be applied in agriculture.

#### 4.2.2. Salt Stress

Many plant RLK genes are involved in the response to salt stress. *OsSIT1* encodes a LecRLK, which mediates ethylene production and salt-induced ethylene signal transduction through the phosphorylation of MPK3/6, leading to the production and accumulation of ROS in plants, and negatively regulating resistance to salt stress [95]. *OsSTLK* encodes LRR-RLK, which may positively regulate plant salt tolerance by regulating the ROS scavenging system, Na^+^/K^+^ ratio, and MAPK (Mitogen-activated Protein Kinases) signal pathway [96]. OsSTRK1 is an RLCK, which resists salt stress and oxidative stress by regulating the dynamic balance of H_2_O_2_ in the plant, and the rice yield is not affected under high salt stress when STRK1 is overexpressed in rice [97]. Detailed analysis shows that Catalase C is the substrate of STRK1 and the phosphorylation on Tyr210 represses the accumulation of H_2_O_2_. This makes OsSTRK1 a promising star in molecular breeding. In addition, STRK1 is phosphorylated upon salt stress, implying that there is an unknown kinase acting upstream of STRK1.

#### 4.2.3. Metal Stress

Plants easily absorb various metals, leading to chlorosis, wilting, cell death, and other toxic symptoms. RLK is related to the detoxification of metals in plants. OsWAK11 can be strongly induced by aluminum and copper, and its expression is upregulated by metals [98]. However, the downstream cues are yet to be validated. Trinh et al. showed that the expressions of the domain of unknown function 26 (DUF26), PR5-like receptor kinase (PR5K) and LRK10-L in rice roots were upregulated under cadmium stress [99]. Even though, functional verification is required by using mutants of these RLKs.

#### 4.2.4. Cold and Heat Stress

Cold stress can affect the fluidity of cell membranes and enzyme dynamics, resulting in the reduction in plant photosynthesis, metabolic disorder, disruption of nutrition transport, and ultimately damages to the plants [108]. *GsLRPK* encodes LRR-RLK. The overexpression of GsLRPK in yeast and Arabidopsis enhances cold resistance and increases the expression of cold response genes [100]. *MtCTLK1* is a cold-tolerance gene newly identified from *Medicago truncatula*. Research showed that MtCTLK1 positively regulates cold tolerance through the C-repeat binding factor (CBF) pathway, antioxidant defense system, and proline accumulation [101]. Notably, RLK may also play negative roles in thermal stress. Expression of CaWAKL20 from pepper reduced the tolerance to heat stress in Arabidopsis by reducing response to ABA [109]. These results suggest that RLKs have dual roles in response to temperature change. However, there is a gap in understanding how RLKs sense to environmental temperature and what is the messenger mediating the perception.

## 5. RLK-Mediated Molecular Crosstalk between Plant Growth and Stress Response

The ability of a plant to switch between growth stimulation and repression in adverse circumstances determines its capacity to withstand stress. As was covered in the sections above, strong evidence suggests that RLKs are crucial in regulating the homeostasis between sustained growth and tolerance to environmental stress. It is crucial to comprehend the process by which plants balance growth with adaptation to ensure survival in order to understand how the spatiotemporal changes in RLKs influence growth and plant adaptation to varied environmental stimuli.

Several mechanisms for how RLKs modulate the interplay between developmental control and stress adaptation have been proposed. One of the most effective strategies is RLK-mediated synergistic and antagonistic regulation between plant hormones. For example, BRs and ABA mostly execute opposing physiological roles. Plant fitness in a changing environment is assumed to be fine-tuned through interactions between BR and ABA signals, and RLKs are critical participants in this important regulatory process. Arabidopsis CRK28 plays critical role in the regulation of overall plant growth. Evidence also demonstrated that CRK28 plays a vital role in regulating ABA signaling during germination and the early stages of root growth. Additionally, increasing CRK28 expression in Arabidopsis resulted in an increase in *P. syringae* disease resistance [70,110].

The biological function and molecular mechanism of Arabidopsis BRI1 has been well-characterized. The direct interaction of BR molecules and BRI1 causes the formation of a BRI1-BAK1 heterodimer (Figure 3), which then sets off an intracellular phosphorylation cascade. As a result, the downstream transcription factors BRASSINAZOLE RESISTANT 1 (BZR1) and BRI1-EMS-SUPPRESSOR 1 (BES1) are promoted in transcriptional activity and protein stability, leading to expression of large numbers of BR output genes and thereby regulating a variety of developmental and stress-responsive events in the plant [33]. It is noted that the Arabidopsis BRI1 mutant, despite having growth-defective traits, was found to be more resistant to cold stress than wild-type and transgenic plants that overexpressed BRI1. This may be partially explained by the *bri1* mutant’s increased expression of *CBFs/DREBs* even when there is no cold stress [111]. Growth of a plant is slowed down as a result of stress, and metabolic energy is redistributed. Thus, it is likely that the *bri1* mutant could always be aware of potential stimuli. To combat harmful environmental changes, such as freezing, the capacity to handle stress in advance may be helpful for plants to survive.

Arabidopsis BAM1 and CRK28 also showed an essential role in balancing plant growth and stress adaptation. BAM1 is necessary for meristem activity, early anther development, viral migration, and gene silencing [72]. In addition to the crosstalk and mechanisms mediated by the above examples, the SERK genes also appear to be crucial for plant development under stress. OsSERK2 was found to be important in the formation of embryogenic cells and rice development. Overexpression of OsSERK2 enhanced the resistance to rice blast [112]. However, OsSERK1, a different SERK protein, does not appear to make a significant difference in disease resistance to bacteria or fungi. It is interesting to note that silencing OsSERK1 reduces the leaf angle but has no effect on other agronomic traits like leaf morphology, plant height, or seed set [113], suggesting that altering OsSERK1 expression may be a beneficial tactic for creating rice varieties with higher yields. 

**Figure 4:**
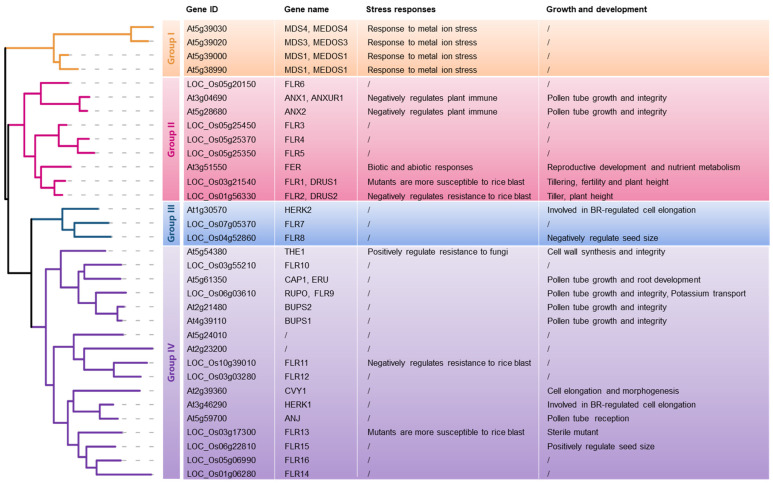
Phylogenetic tree of the CrRLK1L subfamily in Arabidopsis and rice. Phylogenetic tree of full-length amino acid sequences of the CrRLK1L subfamily in Arabidopsis and Oryza was constructed using MEGA 7.1 with the NJ method (all parameters were default values).

In recent decades, great progress has been made in the research on the mechanism of RLKs, among which CrRLK1 has attracted much attention. Members of the CrRLK1L family were first found in the genus *Catharanthus*, so they were named after the Latin word CrRLK1 of the genus. It is characterized by two malectin-like domains, a transmembrane domain and an intracellular Ser/Thr kinase domain [114]. CrRLK1L receptor kinase plays an important role in regulating plant growth and development and coping with various stresses [115,116,117,118,119,120,121,122,123,124,125]. At present, 17 and 20 hypothetical CrRLK1L members have been retrieved from Arabidopsis and rice genomes [126], and some of their functions have been identified (Figure 4).

Arabidopsis FER is the most representative member of CrRLK1L family in functional characterization [118,127,128,129,130]. Rapid alkalization factor (RALF) peptide is the ligand of CrRLK1L. The most reported RALF can induce the rapid alkalization of plant extracellular chambers and regulate a series of plant reactions [131,132,133]. Some proteins are thought to be related to the RALF-FER signal, such as cell wall components and glycosylphosphatidylinositol-anchored proteins (GPI-APs), which directly or indirectly bind to the FER extracellular domain to sense the RALF signal [116]. LORELEI (LRE) is a GPI-AP protein, and LRE-like GPI-AP1 (LLG1) also directly interacts with the FER extracellular domain as a co-receptor to sense RALF signals [134]. In addition to LLG1-FER interactions, LLG2 and LLG3 form different receptor/co-receptor complexes with other CrRLK1L members ANXUR (ANX) and Buddha’s Paper Seal (BUPS) to sense RALF4 and RALF19 peptides. ANX1/2 and BUPS1/2 maintain pollen tube growth by preventing late pollen tube rupture and sperm cell excretion [116]. BUPS1 directly acts upstream of Rho-like GTPase from Plant 1 (ROP1), and rapidly regulates the integrity of its cell wall when the pollen tube appears in the osmotic growth stage. This process requires RALF4 and RALF19 [135].

FER interacts with the cell wall localization protein Leucine-rich repeat extensins3/4/5 (LRX3/4/5) to regulate vacuole expansion during cell elongation [136]. FER also monitors carbon/nitrogen (C/N) balance through an interaction with ARABIDOPSIS TÓXICOS EN LEVADURA6 (ATL6), regulates mRNA selective splicing through phosphorylated RNA binding protein GRP7 (lysine-rich protein 7), regulates plant adaptability and flowering time [119], and promotes protein synthesis and polar cell growth through phosphorylated eIF4E1 [137]. The loss-of-function mutants of *FER* gene are more sensitive to *P. syringae*. RALF23 binds to the extracellular domain of FER and inhibits the formation of immune-related complexes between FER-mediated EF-TU receptors (EFR), FLS2, and its BAK1, thereby negatively regulating the immune response [118,138]. FER carries out immunity processes by promoting the formation of the immune complexes FLS2-BAK1 and EFR–BAK1 and inhibits plant immunity by combining with the RALF1 peptide [118].

*fer* is highly sensitive to salt, cold, heat stress, and ABA, indicating that FER is helpful to maintain plant growth under abiotic stress conditions [115,139,140,141]. Under salt stress, Na^+^ replaces Ca^2+^ in the cell wall and destroys pectin and the cell wall [142]. The function of FER in salt resistance requires the participation of LRX3/4/5. The salt-sensitive phenotype of the *lrx3/4/5* triple mutant is similar to that of the *fer-4* mutant. A group of phylogenetic-related RALF peptides (RALF22/23) interacts with the LRX3/4/5 protein and FER protein. LRX3/4/5 protein and FER protein induced the maturation of RALF peptide under salt stress. The overexpression of RALF phenocopied *fer* and *lrx3/4/5* triple mutants. It was speculated that RALF might promote the cytoplasmic internalization of FER [143]. These results indicate that the LRX3/4/5-RALF22/23-FER module coordinates plant growth and stress response. Salt-induced cell wall damage may be sensed by LRX protein, and then release RALF peptide to promote FER internalization. The LRX3/4/5-RALF22/23-FER module also controls plant growth and salt stress response by regulating the homeostasis of plant hormones (JA, SA, and ABA) [144,145].

In rice, CrRLK1L homologues have been shown to be involved in regulating reproduction [126,146], grain size, and quality [147]. Some OsFLR members also regulate many agronomic traits [119]. In terms of immunity, rice *flr2* and *flr11* mutants are resistant to rice blast without affecting growth. The *flr1* and *flr3* mutants are more sensitive to rice blast [148]. In addition, the soybean pathological mimic mutant 1 (*GmLMM1*) encodes an FER receptor-like protein kinase, which negatively regulates the formation of the FLS2-BAK1 complex induced by flg22, and the mutant shows an enhanced resistance to bacterial and oomycete pathogens [119]. 

## 6. Concluding Remarks and Future Perspectives

RLKs, one of the largest gene families in plants, play critical roles in regulating plant growth and development, signal transduction, stress response, among others. Understanding the function and molecular mechanism of RLKs is helpful for crop genetics and breeding. Though many RLKs have been identified in plant growth, development and stress response, this field of research also faces challenges. As RLKs associate with plasma membrane, novel RLKs are hard to characterized due to the difficulties in isolating plasma membrane protein [149]. In addition, most of the functional studies regarding plant growth are about LRR-RLKs (Table 1), while RLKs function of other subfamilies still need to be explored. Moreover, the exact roles of RLKs may be masked due to their redundancy [150].

Future efforts are needed to gain knowledge of RLKs concerning several aspects. Firstly, although many types of RLKs have been found in plants (especially in Arabidopsis and rice), the research on RLKs’ simultaneous regulation of growth and stress resistance in other plants is relatively few. Secondly, the molecular mechanism of RLKs in plant regulatory networks is still unclear. It is expected to identify upstream and downstream components of RLKs. Recent papers suggested that MAPK cascades and heterotrimeric G proteins acted downstream of RLKs [151], but the possible factors linking MAPK cascades or heterotimeric G proteins are not known. Moreover, the downstream pathways of most RLKs remain elusive, like LURE1 receptors MDIS1-INTERACTING RECEPTOR LIKE KINASE 1 (MIK1), MIK2, POLLEN RECEPTOR LIKE KINASE 6 (PRK6), MALE DISCOVERER 1 (MDIS1) [42]. Protein interactome analysis and yeast two hybrid screening may fish out interesting downstream components of RLKs [10]. Another challenge is the identification of their ligands. RLKs have diverse types of extracellular domains which recognize external signals, and it is reported that over 1000 RLKs genes reside in rice genome [152], implying the ligands of RLKs are largely unknown [42]. The regulation of RLK level is also an important issue. Previous studies have shown that BRI1 can be ubiquitylated by their phosphorylated E3 substrate PUB13 [153]. Whether RLKs abundance or activity can be regulated through other ways is also intriguing. Thirdly, limited energy and nutrients compromise plant growth and defense, and an enhanced stress response usually comes at the expense of crop growth and grain yield. However, little is known about how RLKs cooperatively regulate plant growth and stress response. Given the significance of RLKs’ biological role and the widespread presence of RLK genes in plants, future research into how RLK-mediated plant growth and stress responses interact may yield fresh information that will help to elucidate the underlying molecular mechanisms. 

Crop breeders constantly strive to create new crop varieties with high yield and stress resistance. The precise regulation of plant growth and stress resistance in specific growth stages or tissues is expected to bring a glimmer of hope to solve this problem. The rapid development of genome sequencing and biological information analysis make it possible to identify more RLK genes in different plants. Further study of the biological function and molecular mechanism of RLKs will be beneficial to crop molecular breeding and to cultivate new crop varieties with a strong resistance and high yield.

## Figures and Tables

**Table 1:** Classification of RLKs according to their extracellular domain.

No.	Type of RLKs	The Extracellular Domain of RLKs
1.	Leucine-rich repeat receptor-like kinases (LRR-RLK)	Leucine-rich repeat domain
2.	S-domain receptor-like kinases (SD-RLK)	S-domain
3.	Epidermal growth factor-like kinases (EGF-RLK)	Epidermal growth factor repeat domain
4.	Wall-associated receptor-like kinases (WAK-RLK)	EGF repeat domain-
5.	Lysin motif-type receptor-like kinases (LysM-RLK)	LysM domain
6.	Lectin receptor-like protein kinases (LecRLK)	Lectin domain
7.	Pathogenesis related protein-5 like receptor kinases(PR5K-RLK)	Thaumatin-like domain
8.	Tumor necrosis factor receptor-like protein kinases (TNFR-RLK)	Tumor necrosis factor receptor domain
9.	Cysteine-rich receptor-like kinase (CRKs)	Cysteine-rich domain
10.	Proline-rich extensin-like receptor kinases (PERK-RLK)	Proline-rich extensin-like domain
11.	Catharanthus roseus receptor-like kinase 1-like (CrRLK1L)	Malectin-like domain

**Table 2:** RLKs regulate plant growth and development.

Plant Species	RLKs	Subfamily	Function	Reference
*Arabidopsis thaliana* (Arabidopsis)	CLV1	LRR-RLK	Meristem and flower development	[19,20,21]
	ERfRLK	LRR-RLK	Cotyledon growth and ovule development	[25,26]
	BAM1/2	LRR-RLK	Anther development	[21]
	RPK2	LRR-RLK	Anther development	[21]
	AtVRLK1	PR5K-RLK	Regulates secondary cell wall thickening; up-regulation of AtVRLK1 leads to defects in anther dehiscence	[27]
	AtPERK5/12	PERK-RLK	Necessary for proper pollen tube growth	[28]
	BRI1	LRR-RLK	Regulates cell elongation by mediation of BR signaling	[29,30,31,32,33]
	EMS1	LRR-RLK	Anther development	[34]
*Oryza sativa* (Rice)	OsERL	LRR-RLK	Anther lobe formation	[35]
	TMS10/ TMS10L	LRR-RLK	Redundant control of male fertility under fluctuating temperatures; regulates tapetal degeneration and pollen development	[36]
	OsLecRK5	LecRLK	Regulates callose biosynthesis during pollen development	[37]
	OsLSK1	SD-RLK	Overexpression of OsLSK1 extracellular domain improves panicle architecture and grain yield	[38]
	OsER1	LRR-RLK	Negative regulator of spikelet number per panicle	[39]
	SERK2	LRR-RLK	Overexpression of *SERK2* enhances grain size and salt resistance	[40]
	OsRPK1	LRR-RLK	Negatively regulates root development	[41]
	OsESG1	SD-RLK	Regulates early crown root development and drought resistance	[42]
*Triticum aestivum* L. (Wheat)	TaBRI1	LRR-RLK	Early flowering and seed yield enhancement in Arabidopsis	[43,44,45]

**Table 3:** RLKs involved in biotic stress response and their functions.

Plant Species	RLKs	Subfamily	Biotic Stress Type/Name	Function	Reference
*Arabidopsis thaliana* (Arabidopsis)	CDG1	RLCK	Bacterial disease/*Pseudomonas syringae*	Negatively regulates Arabidopsis pattern-triggered immunity (PTI)	[68]
	AtSERK1/AtSERK2	LRR-RLK	Bacterial disease/*Pseudomonas syringae*	Resistance to bacterial leaf blight and fungal infection	[69]
	CRK28/ CRK29	CRK	Bacterial disease/*P. syringae*	Enhances plant immune responses	[70]
	AtBAK1	LRR-RLK	Fungal diseases/ *Cladosporium fulvum*	Triggers immune signaling to promote plant resistance against pathogens	[71]
	BAM1	LRR-RLK	Viral disease/*Tobacco mosaic virus* (TMV)	Involvement in the early stages of TMV spread and cell-to-cell movement	[72]
	NIK1	LRR-RLK	Viral disease/begomovirus *Cabbage leaf curl virus*; Bacterial disease /*P. syringae* DC3000 and ES4326	Positively regulates plant antiviral immunity; Negatively regulates plant of antibacterial immunity	[73]
*Oryza sativa* (Rice)	rrsRLK	RLCK	Bacterial disease/*Xanthomonas oryzae* pv. *oryzae* (*Xoo*)	Δ*rrsrlk* resistant to bacterial leaf blight in Rice	[74]
	Pi65	LRR-RLK	Fungal diseases/*Magnaporthe oryzae*	Overexpression of Pi65 enhanced rice blast resistance	[75]
	SDS2	SD-RLK	Fungal diseases/*M. oryzae*	SDS2 overexpression enhanced resistance to *M. Oryzae*	[76]
	OsSOBIR1	LRR-RLK	Viral disease/Rice black-streaked dwarf virus(RBSDV)	Regulates the PTI response and rice antiviral defense to RBSDV	[77]
	OsLRR-RLK1	LRR-RLK	Herbivore attack/striped stem borer (SSB) *Chilo suppressalis*	Against the chewing herbivore SSB	[78]
	OsLRR-RLK2	LRR-RLK	Herbivore attack/brown planthopper (BPH, *Nilaparvata lugens*)	Negatively regulates the resistance of rice to BPH	[79]
*Triticum aestivum* L. (Wheat)	TaXa21	LRR-RLK	Fungal diseases/*Puccinia striiformis* f. sp. *tritici*	Positive regulator of wheat High-temperature seedling-plant resistance to *P. Striiformis* f. Sp. *Tritici*	[80]
	TaCRK10	CRK	Fungal diseases/*P. striiformis* f. sp. *tritici*	High-temperature seedling-plant resistance to stripe rust caused by fungal pathogen *P. striiformis* f. Sp. *Tritici*	[81]

**Table 4:** RLKs involved in abiotic stress response and their functions.

Plant Species	RLKs	Subfamily	Biotic Stress Type/Name	Function	Reference
*Arabidopsis thaliana* (Arabidopsis)	RLK7	LRR-RLK	Drought stress	Regulates immune responses and stomatal closure	[90,91]
	RPK1/BAK1	LRR-RLK	Drought stress	Positively regulates ABA-induced stomatal closure	[92]
*Oryza sativa* (Rice)	LP2	LRR-RLK	Drought stress	Negative regulator in drought response	[93]
	HSL3	LRR-RLK	Drought stress	Regulates stomatal closure and drought stress response	[94]
	OsSIT1	LecRLK	Salt stress	Negatively regulates salt sensitivity	[95]
	OsSTLK	LRR-RLK	Salt stress	Positive regulator of salt stress tolerance	[96]
	OsSTRK1	RLCK	Salt stress	Positively regulates salt and oxidative stress tolerance	[97]
	OsWAK11	WAK-RLK	Metal stress/aluminum and copper	Regulates resistance to aluminum and copper	[98]
	LRK10-L	PR5K-RLK	Metal stress/cadmium	Regulates chromium stress	[99]
	DUF26	CRK	Metal stress/cadmium	Regulates chromium stress	[99]
*Glycine soja* (Soybean)	GsLRPK	LRR-RLK	Cold stress	Positive regulator to cold stress tolerance	[100]
*Medicago truncatula*	MtCTLK1	LRR-RLK	Cold stress	Positive regulates cold tolerance	[101]

## Data Availability

No new data were created or analyzed in this study. Data sharing is not applicable to this article.
